# Clinical features and surgical selection in colitis-associated colorectal cancer with ulcerative colitis

**DOI:** 10.1186/s12893-023-02160-x

**Published:** 2023-08-28

**Authors:** Ryuichi Kuwahara, Hiroki Ikeuchi, Kurando Kusunoki, Tomohiro Minagawa, Yuki Horio, Kei Kimura, Kozo Kataoka, Naohito Beppu, Masataka Ikeda, Motoi Uchino

**Affiliations:** Department of Gastroenterological Surgery, Division of Inflammatory Bowel Disease Surgery, Hyogo Medical University, Nishinomiya, Japan

**Keywords:** Ulcerative colitis, Surgical treatment, Cancer, Dysplasia, Refractory

## Abstract

**Purpose:**

The aim of this study was to compare the clinical characteristics of ulcerative colitis (UC) patients who underwent surgery for cancer/dysplasia with those who underwent surgery for refractory disease and to discuss the preoperative preparation for successful hand-sewn IPAA.

**Methods:**

Patients who underwent surgery for UC between January 2014 and December 2021 at Hyogo Medical University were included in the study. A total of 443 UC surgical cases were included in the study, which comprised 188 cancer/dysplasia patients and 255 refractory patients. Clinical records were compared retrospectively.

**Results:**

The proportion of surgical UC cases with cancer/dysplasia has been on the rise, accounting for approximately 40% in recent years. The duration of disease (months) was 186 (2–590) in the cancer/dysplasia group and 48 (1–580) in the refractory group (*p* = 0.02). UC severity (mild/moderate/severe) was 119/69/0 in the cancer/dysplasia group and 18/157/80 in the refractory group (*p* < 0.01). The four nutrition factors of weight (55.2 (32.7–99.6) kg: 49.9 (20.3–85.2) kg), body mass index (21.0 (13.9–32.5) kg/m2: 18.3 (11.4–34.1)kg/m2), serum albumin level (4.3 (2.7–5.0)g/dl: 3.4 (1.4–5.2)g/dl) and prognostic nutrition index (49.2 (33.2–61.2): 40.9 (17.4–61.1)) were significantly higher in the cancer/dysplasia group (*p* < 0.01). The degree of obesity was also significantly higher in the cancer/dysplasia group (*p* < 0.01).

**Conclusion:**

UC patients with cancer/dysplasia were more likely than refractory patients to have mild inflammation; they also had a longer duration of UC disease and better nutritional status.

## Introduction

The number of patients with ulcerative colitis (UC) is increasing worldwide [1], and the number of Japanese patients with UC is estimated to have exceeded 200,000 [2].

In the past, the majority of cases were refractory to surgical indications [3], but recent advances in medical therapy, especially with the advent of tacrolimus and biologic agents, have increased treatment options. As a result, the number of cases in which surgery can be avoided is increasing [4, 5]. While the number of refractory UC cases in which surgery can be avoided has increased, the number of cases that become cancerous due to long-term inflammation has also increased [6]. Eaden et al. reported that the cancer incidence rate of UC is 2.1% at 5 years, 8.5% at 10 years, and 17.8% at 20 years of disease duration [7]. Given continuing advances in medical therapy, the number of patients with cancer dysplasia can be expected to increase.

The basic surgical treatment for UC is restorative proctocolectomy (RPC) with hand-sewn/stapled ileal J-pouch anal anastomosis (hand-sewn IPAA), and there is a certain consensus regarding its safety [8]. At our institution, hand-sewn IPAA is the basic technique, but some institutions use stapled IPAA, which preserves the mucosa of the anal canal, as the standard technique from the viewpoint of preserving sphincter function after surgery [9]. There are differences among facilities. In cases where the indication for surgery is rectal cancer/dysplasia, hand-sewn IPAA is recommended due to the risk of recurrence of cancer from the residual anal canal mucosa [10, 11]. However, among cases in which cancer/dysplasia is an indication for surgery, there are a few in which hand-sewn IPAA is difficult because the J-pouch does not reach the anus due to obesity. In fact, a case of dysplasia from the residual anal canal mucosa was reported [10]. The inability to perform hand-sewn IPAA is a clinically important problem, especially in patients at high risk of cancer in the anal transitional zone.

The purpose of this study was to compare the clinical characteristics of cases operated on for cancer/dysplasia with those operated on for refractory disease and to discuss the preoperative preparation for successful hand-sewn IPAA.

## Materials and methods

### Patients and data collection

Patients who underwent surgery for UC between January 2014 and December 2021 at Hyogo Medical University were included in the study. Overall, 604 patients were included, 188 of which were operated on due to cancer/dysplasia and 255 of which were operated on due to refractory disease. Each study factor (patient characteristics, duration of disease, preoperative medical treatments, surgical indications, preoperative nutrition status) was compared retrospectively. Emergency surgery cases were excluded.

### Definitions

The diagnosis of UC was based on recognized radiological, endoscopic, and histopathological criteria. UC disease activity is assessed based on clinical features using the criteria of Truelove and Witts [12].

Patients were classified according to the categories proposed by the International Obesity Task Force as nonobese (body mass index [BMI] < 25.0 kg/m^2^), obese I (BMI 25.0-29.9 kg/m^2^), and obese II (BMI≧30 kg/m^2^).

Blood parameters were also retrospectively obtained from the clinical records, including C-reactive protein (CRP) levels, white blood cell (WBC) counts, serum albumin levels, hemoglobin (HGB) levels, and TLC just before initial surgery. Furthermore, we reviewed Onodera's prognostic nutritional index (O-PNI) values, which were calculated as 10 × serum Alb (g/dL) + 0.005 × TLC [13].

### Statistical analysis

All statistical analyses were carried out using JMP ver. 15 (SAS Institute, Inc., Cary, North Carolina, USA). Each variable was compared with the Pearson χ^2^ test, Student’s *t* test, or Mann–Whitney *U* test. Statistical significance was set as a p value less than 0.05.

### Ethical considerations

All study protocols were approved by the Institutional Review Board of Hyogo Medical University (No. 4210).

## Results

### Patient characteristics

Table 1 shows the number of cases of cancer/dysplasia that resulted in surgery in our hospital. In 2012, the proportion of cancer/dysplasia cases was approximately 20%, but in the past few years, the number has been increasing to 30-40%. The patients’ clinical characteristics are shown in Table 2. The sex (male/female) ratios were 112/76 in the cancer/dysplasia group and 174/81 in the refractory group (*p*=0.07). The ages at surgery (years; median (range)) were 54 (25-81) years in the cancer/dysplasia group and 47 (7-90) years in the refractory group (*p*<0.01). The duration of disease (months) was 186 (2-590) in the cancer/dysplasia group and 48 (1-580) in the refractory group (*p*=0.02). UC severity (mild/moderate/severe) was 119/69/0 in the cancer/dysplasia group and 18/157/80 in the refractory group (*p*<0.01).

**Table 1:** Number of cases of cancer/dysplasia that resulted in surgery

**Year**	**Cases**	**Cancer/dysplasia**	**Frequency(%)**
**2012**	**91**	**19**	**20.9**
**2013**	**92**	**21**	**22.8**
**2014**	**94**	**27**	**28.7**
**2015**	**96**	**30**	**31.3**
**2016**	**94**	**24**	**25.5**
**2017**	**70**	**23**	**33.0**
**2018**	**86**	**23**	**27.7**
**2019**	**63**	**22**	**34.9**
**2020**	**50**	**22**	**44.0**
**2021**	**55**	**19**	**34.5**

**Table 2:** Patient characteristics

	**Cancer/dysplasia** **(** ***n*** ** = 188)**	**Refractory** **(** ***n*** ** = 255)**	***p***
**Sex (Male/Female)**	**112/76**	**174/81**	**0.07**
**Age at Surgery (y)**	**54 (25–81)**	**47 (7–90)**	**< 0.01**
**Duration of disease (mo)**	**186 (2–590)**	**48 (1–580)**	**< 0.01**
**UC severity** **(Mild/Moderate/severe)**	**119/69/0**	**18/157/80**	**< 0.01**

Continuous variables are indicated as the mean and range

### Preoperative medical treatments

The preoperative medical treatments are shown in Table 3. The total steroid dose (mg; median (range)) was 1,000 (0-100,000) in the cancer/dysplasia group and 3,190 (0-150,000) in the refractory group (*p*=0.97). The daily steroid doses (mg) were 0 (0-40) in the cancer/dysplasia group and 0 (0-60) in the refractory group (*p*<0.01). Immunosuppressive drugs were used in 63 (33.5%) patients in the cancer/dysplasia group and 208 (81.6%) in the late group (*p*<0.01). Leucocyte apheresis was used in 40 (21.3%) patients in the cancer/dysplasia group and 122 (47.8%) in the refractory group (*p*<0.01). Antitumor necrosis factor agents were used in 49 (26.1%) patients in the cancer/dysplasia group and 188 (73.7%) in the refractory group (*p*<0.01). The four factors of daily steroid doses, immunosuppressive drug use, leucocyte apheresis use and antitumor necrosis factor agent use were significantly higher in the refractory group.

**Table 3:** Preoperative medical treatment

	**Cancer/dysplasia** **(** ***n*** ** = 188)**	**Refractory** **(** ***n*** ** = 255)**	***p***
**Steroid (total dose)**	**1,000 (0–100,000)**	**3,190 (0–150,000)**	**0.97**
**Steroid (daily dose)**	**0 (0–40)**	**0 (0–60)**	**< 0.01**
**Immunosuppressive drugs (%)**	**63 (33.5)**	**208 (81.6)**	**< 0.01**
**Leukocytapheresis (%)**	**40 (21.3)**	**122 (47.8)**	**< 0.01**
**Anti-TNFα agents (%)**	**49 (26.1)**	**188 (73.7)**	**< 0.01**

Continuous variables are indicated as the mean and range

Categorical data are numbers with percentages in parentheses

### Preoperative nutritional status

Table 4 shows the preoperative nutritional status. The weight (kg; median (range)) was 55.2 (32.7-99.6) kg in the cancer/dysplasia group and 49.9 (20.3-85.2) kg in the refractory group (*p*<0.01). The height (m; median (range)) was 1.63 (1.43-1.90) in the cancer/dysplasia group and 1.63 (1.15-1.87) in the refractory group (*p*=0.32). The body mass index (median (range)) was 21.0 (13.9-32.5) in the cancer/dysplasia group and 18.3 (11.4-34.1) in the refractory group (*p*<0.01). The serum albumin level (g/dl; median (range)) was 4.3 (2.7-5.0) in the cancer/dysplasia group and 3.4 (1.4-5.2) in the refractory group (*p*<0.01). The prognostic nutrition index (median (range)) was 49.2 (33.2-61.2) in the cancer/dysplasia group and 40.9 (17.4-61.1) in the refractory group (*p*<0.01). The four factors of weight, body mass index, serum albumin level, and prognostic nutrition index were significantly higher in the refractory group.

**Table 4:** Preoperative nutritional status

	**Cancer/dysplasia** **(** ***n*** ** = 188)**	**Refractory** **(** ***n*** ** = 255)**	***p***
**Weight (kg)**	**55.2 (32.7–99.6)**	**49.9 (20.3–85.2)**	**< 0.01**
**Height (cm)**	**1.63 (1.43–1.9)**	**1.63 (1.15–1.87)**	**0.32**
**Body mass index**	**21.0 (13.9–32.5)**	**18.3 (11.4–34.1)**	**< 0.01**
**Serum albumin level (g/dL)**	**4.3 (2.7–5.0)**	**3.4 (1.4–5.2)**	**< 0.01**
**Prognostic nutrition index**	**49.2 (33.2–61.2)**	**40.9 (17.4–61.1)**	**< 0.01**

Continuous variables are indicated as the mean and range

### Degree of obesity

Table 5 indicates the degree of obesity in each group. One hundred sixty-six (166) patients were nonobese (BMI < 25.0 kg/m2) (88%) in the cancer/dysplasia group, and 244 (96%) were nonobese in the refractory group. The proportion of obese I patients (BMI 25.0–29.9 kg/m2) was 10% (18) in the cancer/dysplasia group and 4% (10) in the refractory disease group. The proportion of obese II patients (BMI ≥ 30 kg/m2) was 2% (4) in the cancer/dysplasia group and 0% (1) in the refractory disease group. The degree of obesity was significantly different between the two groups (*p* < 0.01).

**Table 5:**
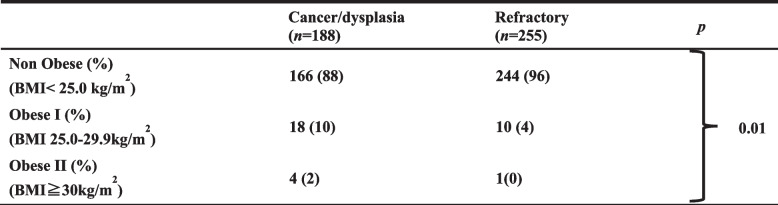
Degree of obesity

Categorical data are numbers with percentages in parentheses

*BMI* body mass index

### Details of cancer cases

In this study, 141 were cancer cases and 47 were dysplasia cases. Of the 141 cancer cases, 79 (56%) underwent surveillance colonoscopy, while 62 (44%) were found incidentally. The Stage (0/I/II/III/IV) of the 141 cancer cases included in this study was 43/45/27/12/11. None of the patients had preoperative interventions such as chemotherapy or radiation therapy. The number of cases in which preoperative endoscopic resection was attempted was 78/36/8/13/3/3 for tissue (tub1/tub2/por/muc/sig/etc.). The other 3 cases were 2 neuroendocrine carcinomas and 1 carcinosarcoma. The age at onset (median(range)) of the 141 cancer cases was 53(24–80) months, and the time from UC onset to cancer diagnosis was 60.5(6.5–114.2) months.

## Discussion

This study showed that patients with cancer/dysplasia had conditions that were less severe, had a longer disease duration, and had better nutritional status than refractory patients. The following is a discussion of how these results can be used clinically.

The ECCO Guidelines recommend screening for patients with UC with a disease duration of more than 6–8 years [14]. In Japan, screening is also recommended for patients who have had UC for more than 8 years [15]. The detection rate of cancer is thought to be increasing as surveillance becomes more widespread. On the other hand, there are some problems with UC, such as the fact that many cases have inflammation of the mucosa in the background, making detection difficult, and that an established surveillance method is lacking. In recent years, there have been reports comparing random biopsy with targeted biopsy [16], and if the quality of surveillance improves in the future, the number of cancer/dysplasia cases may increase. As the quality of surveillance improves, the number of cancer/dysplasia cases may increase, and the number of surgical cases is expected to increase accordingly. Therefore, we believe that this study, in which the characteristics of cancer/dysplasia cases are analyzed from a surgical perspective, is important for future clinical practice.

Hand-sewn IPAA and stapled IPAA, the basic techniques for UC, are standard worldwide. The advantage of hand-sewn IPAA is that the entire colonic mucosa can be resected, while the disadvantage is soiling due to internal anal sphincter dysfunction. Hand-sewn IPAA involves difficult to perform mucosectomy and hand-sewn anastomosis. In stapled IPAA cases, sphincter function is preserved, but the mucosa of the anal canal remains. Therefore, some centers use stapled IPAA as the basic technique from the viewpoint of anastomosis risk and preservation of sphincter function.

We have reported that the incidence of cancer and dysplasia from the residual anal canal mucosa after stapled IPAA is approximately 3.4% (3/88) [11]. We have also reported that 6.4% (14/220) of patients who underwent hand-sewn IPAA with mucosal resection for UC-related cancer had cancer in the anal canal mucosa and that the risk factors were multiple cancers and rectal cancer [11]. The ECCO guidelines also note that hand-sewn IPAA is a reasonable technique in such cases because lower rectal cancer is a risk factor for carcinogenesis from the mucosa of the anal canal [17]. Regarding the localization of cancer/dysplasia, it has been reported that rectal and sigmoid colon carcinomas occur more commonly in patients with UC due to the continuous spread of inflammation from the rectum to the oral side. In the Japanese real-world surgical resection case report, sigmoid colon cancer accounted for 20%, and rectal cancer accounted for 50% [18]. In a large proportion of cases from Canada (36.5%; 443/1213), rectal cancer was reported [19].

Based on the above, hand-sewn IPAA may be more often indicated for patients with cancer/dysplasia in UC, depending on the localization of the tumor, than for patients with refractory UC. A potential problem is obesity. In recent years, a number of new drugs have been introduced, greatly increasing medical treatment options. This has led to an increase in the number of UC cases with chronic inflammation and an increase in cancer/dysplasia cases due to the avoidance of surgery. These patients are in good nutritional condition; in this study, serum albumin levels and BMI were significantly higher than in refractory cases. Some patients had a BMI over 30. In these patients, hand-sewn IPAA may not be performed because of the thick tissue of the mesentery and the poor accessibility of the ileal pouch to the anastomotic site. As a result, preservation of the mucosa of the anal canal, which is at high risk for cancer, may be necessary. In this case, rectal amputation and permanent ileostomy may be needed. To prevent such situations, daily weight control may be important, especially in patients with long disease duration and anorectal inflammation. In fact, in the present study, we have experienced cases in which hand-sewn IPAA was possible with preoperative weight control. In cancer patients, it is not possible to lose weight for a long period of time before surgery, so it is important to maintain a certain degree of weight control on a daily basis. In addition, it has been reported in the field of colorectal cancer that high BMI or obesity is a risk factor for complications such as operation time, blood loss, and death [20–22]. It is desirable to keep weight under control from the viewpoint of safe cancer surgery.

With the advent of newer drugs, the number of refractory cases is likely to decrease, which will equate to an increase in the number of cancer/dysplasia cases. Most patients with UC who have relatively well-controlled inflammation are greatly surprised and anxious when they are told that they need surgery for cancer/dysplasia. To prevent this from happening in advance, it may be important to explain the surgical procedure to patients undergoing surveillance. To make the surgical outcome more desirable for patients, it is important to manage nutritional status from the UC follow-up period in patients with rectal inflammation and multiple dysplasia lesions. In patients with a preoperative BMI over 30, surgery after weight loss is an option, taking into consideration the progression of the cancer.

There are several limitations to this study. First, this is a single-center, retrospective study. Second, Hyogo Medical University is one of the few institutions in Japan with an IBD center. Therefore, there is bias because more patients with severe IBD are seen there. As a result, the postoperative mortality rate may be higher. Second, most of the patients in this study period underwent open surgery, and the rate of laparoscopic surgery was low.

## Conclusion

UC with cancer/dysplasia cases were more likely to involve mild inflammation than refractory cases, and such patients also had a longer duration of UC disease and better nutritional status.

## Data Availability

The datasets used and/or analysed during the current study available from the corresponding author on reasonable request.
